# The Po Delta is restarting progradation: geomorphological evolution based on a 47-years Earth Observation dataset

**DOI:** 10.1038/s41598-018-21928-3

**Published:** 2018-02-22

**Authors:** A. Ninfo, P. Ciavola, P. Billi

**Affiliations:** 1 University of Ferrara, Ferrara, Italy; 2 University of Tottori, Tottori, Japan

## Abstract

From the 1950s, the Po delta, one of the largest anthropogenic world deltas, has been subjected to a fast degradation and shoreline retreat due a marked reduction of sediment supply, mainly controlled by human impacts/factors, including subsidence. Through the interpretation of satellite images, coupled with the analysis of the flow discharge, and of the annual frequency of marine storms, we show that recently (>2010) the Po River has resumed delta progradation, especially in its northern portion. This happens after decades of erosion, followed by alternating regrowth and degradation phases, indicating conditions of substantial stability (1970–2000). Today the delta shows aggradation of new mouth-bars at the main distributary mouth, a clear evidence of active constructive processes. The ongoing trend marks a countertendency compared to many deltas worldwide.

## Introduction

**Figure 1:**
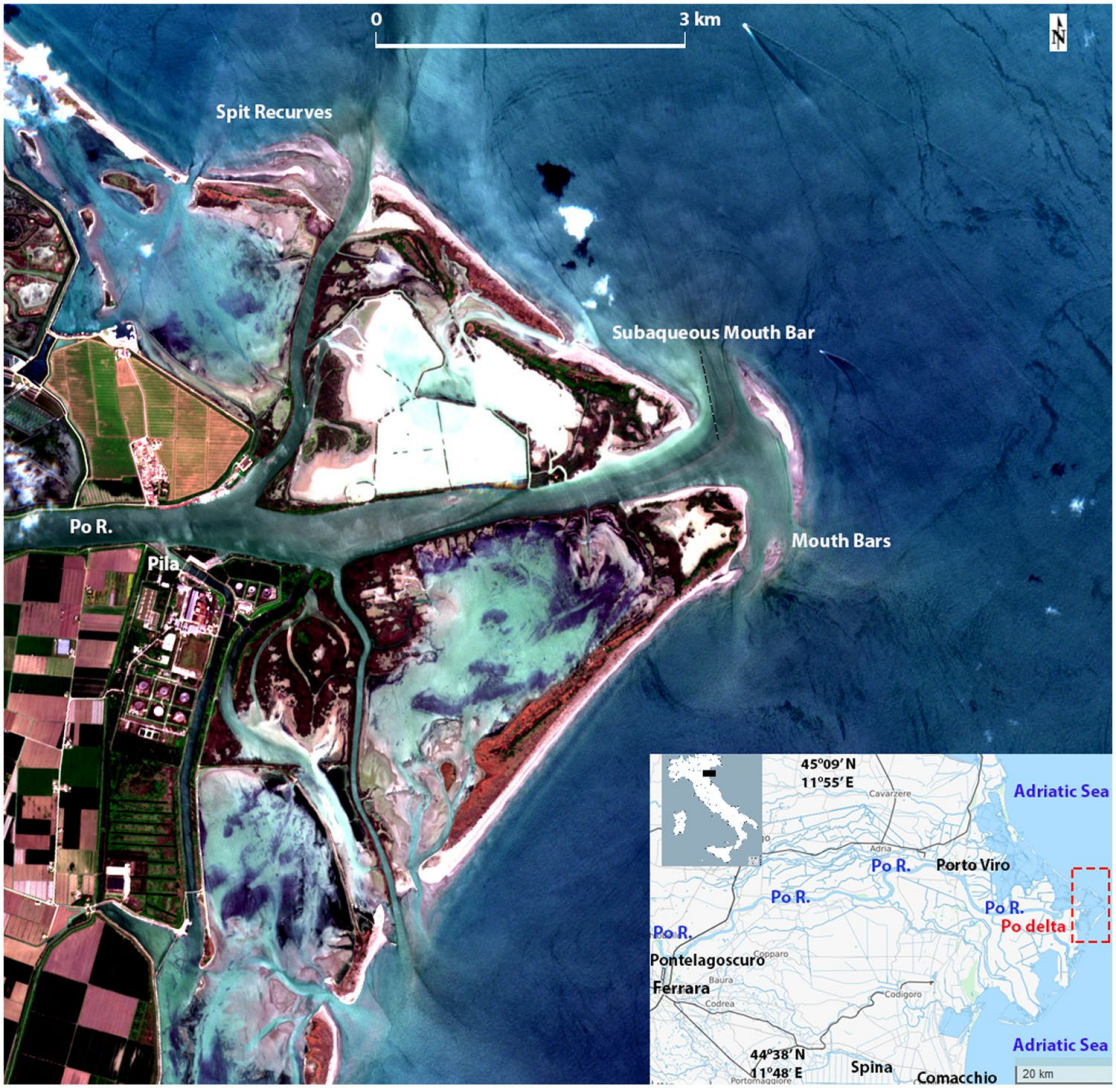
Sentinel 2a scene (L1C_T32TQQ_A009743_20170504T101349), acquired on 4 May 2017, visible false color composition (B432+ Gaussian_stretch, 10 m cell size). In this normal low-tide image the prograding landforms are clearly shown: notice the mouth bars in front of the main Pila mouth, and on the secondary distributary channel (N oriented) a series of three spit recurves. The satellite image was processed by the authors using Copernicus Sentinel 2017 data (L1C level of processing), downloaded from https://eros.usgs.gov/about-us/data-citation under EU open access policy (https://sentinel.esa.int/web/sentinel/sentinel-data-access). The map in the inset was obtained from Open Street Map under Open Data Commons Open Database License (ODbL) V1.0: https://opendatacommons.org/licenses/odbl/. The location map of Italy was obtained from http://d-maps.com/carte.php?num_car=14528&lang=it under Open Access policy http://d-maps.com/conditions.php?lang=it.

The Po is the largest Italian river, draining an area of 70,091 km^2^ and flowing for 650 km (Fig. 1). The Po River sediment supply to the Adriatic Sea has generated one of the largest Mediterranean deltaic system (emerged delta area ~700 km^2^). The Po delta is considered relatively young (~4–5 ky)^1^ and can be described as a delta dominated by both natural and anthropogenic process^2^. Historically, the delta developed through different branches, the position of which was quite different from the present one. The Po delta evolution has been driven by natural fluvial processes (floods/avulsions) and by a growing anthropogenic influence (e.g. engineering works and extensive land reclamation)^2^.

The delta progradation near the current position started in the early 17^th^ century, following massive hydraulic works such as the ‘Porto Viro” bypass. The works were made by the Venetian Republic in 1604 to divert the delta southwards in order to prevent the sedimentation generated by the northern branches into the Venice lagoon^3^. The recent human influence (e.g. dredging near the mouths, artificial consolidation of spits/beaches through sand feeding, channel bank protection, man-induced subsidence, etc.) has been shaping the delta and it is still a dominant controlling factor. Most likely, the Po delta can be considered among the best examples of man-made (anthropogenic) delta in the world^4^.

The historical maximum sedimentation (and progradation) rates were associated with the Little Ice Age climatic transition^1^, whereas during the 20^th^ century, the delta progradation slowed down.

The delta reached its maximum extension between 1930s–1940s; thereafter a significant retreat started at a rate of tens of meter per year, with a negative peak in the 1954–1978 interval^2^. This reduction in sediment yield, generally observed in all Italian rivers after World War II, was caused by different factors including bed material exploitation, dam construction, landslide stabilization, and a progressive reduction of agriculture on low mountain and hilly areas which was commonly followed by reforestation or by a natural regrowth of bush and low forest^5^. In the same period, a marked subsidence, due to extraction of methane and water from shallow aquifers, occurred. These actions resulted in a halt of progradation and a very rapid shrinkage of the deltaic body, as observed in other large delta systems around the world (e.g. Colorado, Nile, Yangtze and Yellow River)^6^. The Po delta, therefore, turned into a fragile and vulnerable landscape, exposed to human modification and climate change impacts like sea level rise and coastal flooding^7^.

The post-1950 reduction in sediment supply generated an overall retreat of the coastline and of all the delta outlets, which was particularly severe in the Po di Pila mouth and the northern part of the delta (Fig. 1). According to previous studies (e.g.^3^), the Po di Pila mouth erosion started in the 1950s and proceeded till today. The Pila mouth is estimated to deliver at least 70% of the whole Po sediment load^8,9^. The sediment input to the delta mouths was modulated also by the fast artificial subsidence experienced by the channel beds during the second half of the 20^th^ century^10^ and caused by fluid withdrawal and by the changing flow proportions between the different distributary channels in consequence of river regulation and water pumping for irrigation.

This part of the delta is therefore ideal to test whether the decreasing trend in sediment supply is still reflected by coastal retreat. Thanks to more than 40 years of satellite Earth Observations (EO), we investigated the coastal changes occurred in the Po Delta during the last phase of general reduction in sediment supply (1970–2000). Moreover, the recent enhanced satellite capabilities (e.g. Sentinel 2) permit to focus, in particular, on the most recent and on the current geomorphological evolution of the Po delta margins.

## Methods

While for the period 1950 onwards researchers had to rely on indirect sources like a not frequently updated cartography and sparse aerial photography, remotely sensed data offers a higher frequency dataset of direct observation.

With the technological progress of scientific optical satellite imaging and access policy, large public datasets are now available, starting from the 1970s through the Landsat missions^1,2^ and, from summer 2015, through the Copernicus Program with the Sentinel-2a satellite, followed, in march 2017, by the Sentinel-2b, which allowed an almost continuous monitoring of the geomorphological evolution of the most active Po delta margins.

**Figure 2:**
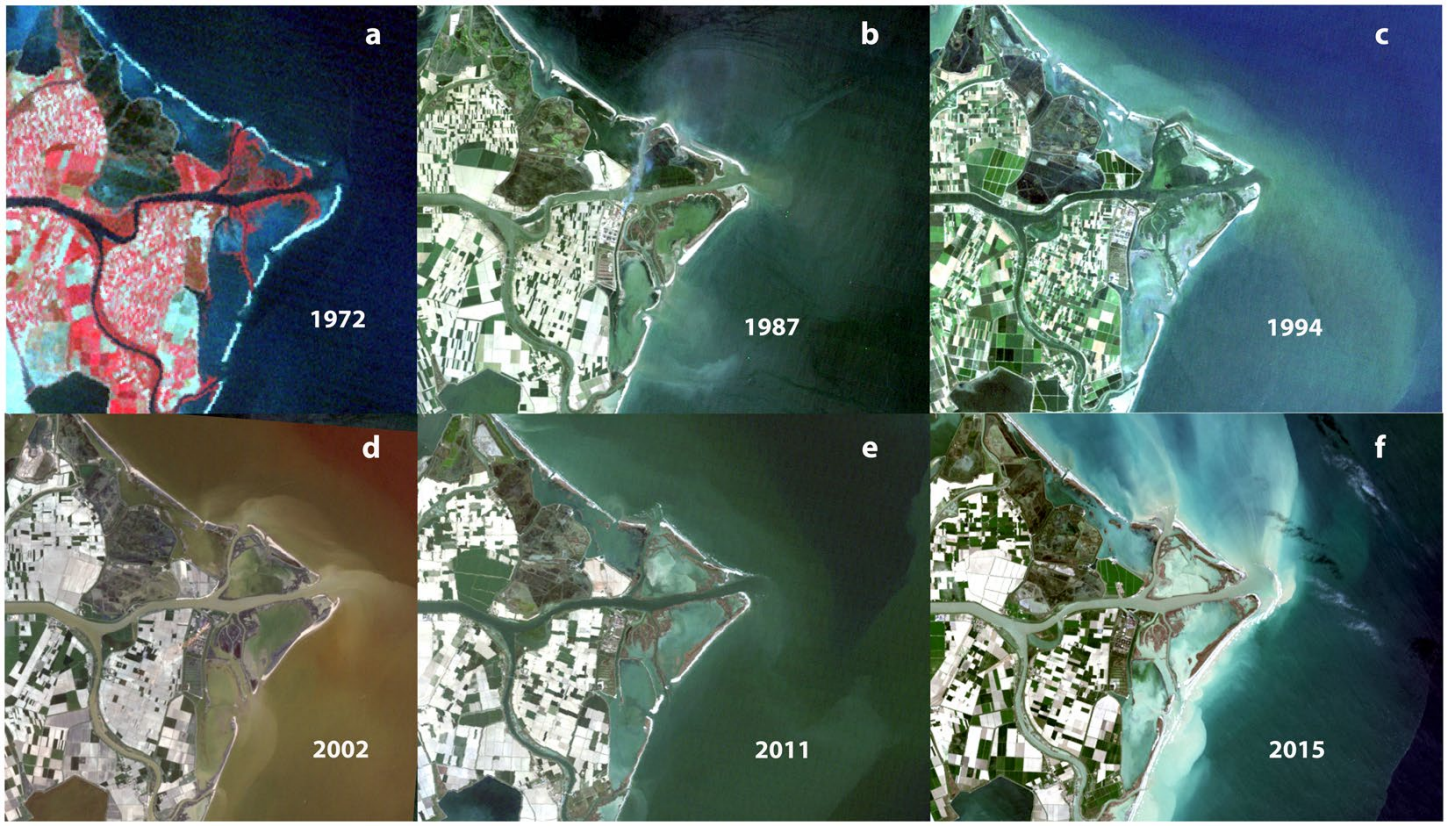
Landsat satellite images: (**a**) L1MSS-12 August 1972 (60 m); (**b**) L5TM 8 May 1987 (30 m); (**c**) L5TM 14 July 1994 (30 m); (**d**) L7ETM 23 April 2002 (30 m); (**e**) L5TM 17 April 1991 (30 m); (**f**) L8OLI 3 April 2015 (30 m). Landsat images were obtained by processing datasets downloaded from the Glovis USGS server (https://glovis.usgs.gov) under open-access policy https://eros.usgs.gov/about-us/data-citation.

Compared with Landsat 8, Sentinel-2 has a better temporal resolution (e.g. it can archive on average at least one cloud free image every week), a better spatial resolution on VIS and NIR (10 m vs 30 m of Landsat) and a good radiometric capability (12 bit depth like the Landsat 8). For this study, images taken during extremely high or low tidal levels were not considered because they could give false information on the position of the shoreline. All the other images were considered acceptable, considering that in the Adriatic Sea the tide range is very limited (0.2–0.8 m). Moreover, some images, acquired with visible wave crests near the coast, underlined the presence of subaqueous landforms (e.g. Fig. 2f), that could be identified by the breaking wave patterns.

To maximize the geometric resolution, the 10 m visible bands of Sentinel 2 were composed in the following order, 4(665 nm) 3(560 nm) 2(490 nm). The false color compositions were digitally enhanced applying a *contrast stretch*, based on the Gaussian distribution of the pixel image values, to emphasize the landform visibility (Fig. 1).

In order to get complementary information on the river delta dynamics, flow measurements carried out by the Po River Authority were obtained for the gauging station of Pontelagoscuro (Fig. 1), approximately 70 km inland and 40 km upstream of the deltaic system apex. The validated data span the 1925–2015 interval, with some gaps around WWII and in recent years. As suspended sediment transport measurements were stopped in 1984, these data were not used. As an alternative, a newly defined effective flow discharge (*Q*_*k*_) was considered a good proxy of sediment yield. In a large river like the Po, sediment yield is controlled by a number of factors, the most important of which are sediment supply and flood frequency. The most downstream reach of the Po river, between the flow gauge of Pontelagoscuro, near Ferrara (Fig. 1) and the mouth, is devoid of tributaries, thus it can be considered a buffer reach in which sediment undergoes a repeated deposition and entrainment conditions. This implies that the river mouth response to the variability of sediment yield is not immediate and a certain time lag should be expected. Furthermore, the sequence of flows with very different discharges may play a very important role in transferring the river bed material to the mouth. Though an individual exceptional flood can entrain, transport and deliver a large quantity of bedload, phases with a number of higher-than-average floods can also be effective in the actual transfer of more or less large quantities of sediment to the mouth. The discharge exceeded 10 days a year (*Q*_*10d*_) is a useful datum that may well represent the fraction of the largest flows, i.e. the most effective for bedload transport. Though yearly variable, Q_10d_, is in fact close to the threshold conditions for bed material entrainment in sandbed rivers of the northern Adriatic coast^11^; moreover, that is the lowest duration flow data available in the official data records for the rivers of the region. On the base of yearly data, as the discharge exceeded 10 days a year (*Q*_*10d*_) is close to the maximum discharge (*Q*_*max*_), and the higher is *Q*_*max*_ the larger amount of sediment is likely to be delivered at the mouth. These two characteristic discharges were combined to obtain a sediment transport capability discharge (*Q*_*k*_) in the following from:

$${Q}_{k}={Q}_{max}\,{Q}_{10d}/({Q}_{max}-{Q}_{10})$$

**Figure 3:**
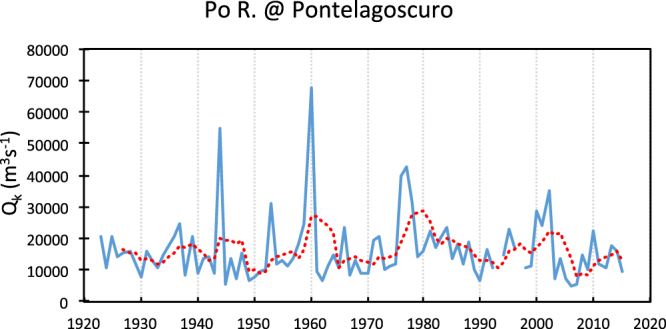
Sediment transport capability, effective discharge (*Q*_*k*_) time series. It shows that in the last 50 years only three periods of potential high sediment flux occurred around 1960, 1976–1977 and 2000–2002. The interval between the last two peaks, 26 years, is the largest of the time series and coincides with the phase of higher retreat rates of the delta. The red dotted line is the five-years moving average.

*Q*_*k*_ was found to well explain the variability of the sediment yield of the Po R. on the base of the data published until 1984. A time series of *Q*_*k*_ yearly values was constructed and plotted in the diagram of Fig. 3, where the five-year mobile average (the dotted red line) is also included to emphasize high and low flow intervals.

To assess the last decade of meteo-marine activity, the storm identification was carried out using data from a wave buoy located at Cesenatico (https://arpae.it/sim/?mare/boa), maintained by the Regional Environment Agency of Emilia-Romagna (ARPAe) and located about 100 km south of the Pila mouths. The data of this measuring station were used to analyze the annual frequency and mean duration of representative storms (Table 1).

**Table 1:** Storms identification during the last ten years using data from the Cesenatico buoy station.

Year	Number of storms	Cumulative number of storm hours	Data gaps
2007	10	226	
2008	14	256	
2009	13	161	January-May
2010	13	234	November-December
2011	13	244	October-December
2012	12	194	January-March
2013	16	361	
2014	7	135	February-June
2015	20	401	
2016	17	301	
2017	12	291	July-October

Storm identification is based on the criteria of^15^ and corresponds to a storm threshold of wave height >1.5 m; individual storms are identified by a 3 hours interval with the wave height below threshold; minimum storm duration considered is 6 hours.

### Data availability statement

The authors declare that the data used for this paper are open-access and available from the institutions who own them. Landsat satellite images are available from the Glovis USGS server https://glovis.usgs.gov. Sentinel images are available from the server of the European Space Agency https://sentinel.esa.int/web/sentinel/sentinel-data-access. River Po Hydrologic Data can be requested to the Po River Basin Authority http://www.adbpo.gov.it. Wave data can be downloaded from the ARPAe server https://www.arpae.it/dettaglio_generale.asp?id=3284&idlivello=1625.

## Results

From the 2015–2017 Sentinel-2 images of the Po delta, the growth of a small barrier/lobe island is evident in front of the main Pila mouth. Initially it was observed only in overpasses at low tide, or from patterns in visible suspended sediment and in alterations in the wave crests, for example in January 2015 (Fig. 2f), confirming a fast growth of the mouth bar, after a series of floods occurred between 2013–15^12^. At about the same time, new crescent-shaped mouth bars (spit recurves) aggraded above sea level at the secondary northern Pila mouth. From the summer/winter 2016 the islands and the mouth bars became evident with almost every tide level. This trend seems to continue until present as shown by the May 2017 Sentinel-2a digitally enhanced image (Fig. 1). The growth direction of these active bars is N-NW, most likely driven by SE waves (*Scirocco* wind), i.e. mainly following a longshore drift direction opposite to the dominant northern wind (*Bora*)^9,13^. Also the main channel outlet and the subaqueous mouth bar are oriented N-NW (Fig. 1).

The coastline analysis (1972–2011), based on the interpretation of at least one images every 7–10 years (Fig. 2), shows that the most active margins of the delta have been subjected to some erosion followed by progradation and then again by erosion. That indicates a certain degree of stability, as recently reported by other authors^14^, with some changes in the ‘arrow’ shape of the delta till the end of the 1990s (Fig. 2a–c). During this interval, a partial growth of the secondary Pila mouth was observed, but later it was eroded. At this location, a slow sedimentation started around 2002 and became more evident in 2011, with the formation of the submerged parts of the mouth bars. These landforms became visible in low tide conditions from 2015 and still continue building-up above the sea level (Fig. 1). Since the 1970s the coastline between the two Pila outlets had advanced of 400–500 m, with some zones growing of 100–200 m in the last 6–7 years (Figs 1–2f).

## Discussion

As marginal beaches at the edge of deltaic spits are continuously formed, eroded and re-deposited, in a continuous battle between wave and river dominance, the interpretation of these precarious landforms from satellite imagery must be taken with caution. In the case of infrequent satellite overpasses, accretion in an image may only reflect temporary conditions. However, when image archives include frequent overpasses (as in the Sentinel constellation), the occurrence of a recent uninterrupted trend can be considered as a reliable evidence of delta progradation (Fig. 1). This is what we observed for the two Po di Pila mouths examined in this study, in a way contradicting previous works that describe the delta still in disastrous conditions regarding the sediment feeding by the river^3,5^.

Deltas are complex and dynamic landforms, with a vast submerged zone that is rarely visible in traditional air photography and/or surveyed during monitoring, especially its shallowest parts, where land surveys cannot extend and large survey vessels cannot access. Remotely sensed optical data allow detecting the changes of even the shallowest coastal morphologies (between tide levels ~0.8–1.5 m) but in the past had the limitation of a low frequency of data acquisition and an unsatisfactory on-the-ground resolution. The Sentinel platform revisiting capabilities permit the frequent comparison between images captured at the same tidal level and increase the possibility of finding acquisitions made during conditions of good water transparency, as in Fig. 1. The inability of a deeper water penetration prevented us from exactly pinpointing when nearshore aggradation of the submerged landforms has started, triggering the emersion of the mouth bar. However, it is clear that the underwater aggradation had already been developing for some times (few years 2–4) as it can be seen in Fig. 2e around the secondary Pila northern mouth and in Fig. 2f on the main outlet.

Two main factors generally control delta progradation: longshore sediment transport system and the amount of sediment supply by the river. Furthermore, storm-related erosive marine processes can play a fundamental role in contrasting the mouths progradation and aggradation. Previous works^10^ found no evidence that in the last two to four decades the coastal sediment transport system and the wave activity have changed. As it can be seen in Table 1, in the last decade, the frequency of storms has remained substantially constant, showing only limited deviation within the normal climate variability, also during the years of the fastest coastal progradation (2015–17). This evidence supports the hypothesis that the sediment budget at the Po river mouth has turned positive again propelling coastal accretion at the northern margins of the delta (Pila mouth bars) after a few decades of low yields.

Figure 3 clearly shows that after about 30 years of relatively low potential effective flows, in the 2000–2002 interval the situation was reversed and higher transfer rates of bed sediment to the mouth were likely resumed. It is worth noticing that this is not the only factor that could have contributed to restore a substantial sediment flux to the Po river mouths. In fact, the prohibition of bed material harvesting, defined by law in the late 1980s, could be showing now its positive effects. The sediment infilling of the very many (thousands) weirs in the headwaters may have contributed as well. They were constructed during the last century all across the mountainous and hilly areas of the Po catchment, as torrent control works to contrast slope degradation and river incision. Nowadays most of these weirs have already fulfilled their original purpose and are completely silted-up. With no more sediment being trapped behind them, they no longer hinder the natural transfer of sediment from the catchment slopes to the river network. Moreover, climate change may have played an important role in terms of accelerated glacier and snow melting in the Alps, which increased the spring season discharges of the Po^4^, resulting in higher values of the potential effective discharge (*Q*_*k*_) (Fig. 3). In Fig. 3 also the five-year mobile average has been included in order to point out prolonged periods with higher values of *Q*_*k*_ which may reflect a substantial bedload supply, more effective in contributing to the formation of mouth bars.

In conclusion, the results of this study point out that the Po delta, after a sediment budget crisis extended throughout the first years of the 21^st^ century, has started again to prograde. In the most recent years (>2010) between the Pila mouths the process has been accelerating and now some progradation is taking place as showed by the growth of mouth bars in Fig. 1. This signal cannot be extrapolated to the whole of the delta, as it is relative to only a portion of it. It is probably necessary to consider a longer time span in the future to assess the response of the whole system.

The current trend marks a countertendency compared to many other world deltas^5,6^ and can be summarized as the result of sediment management policy paired by unexpected climate change positive feedbacks (e.g. accelerated spring ice/snow melting). In any case the area still remains highly vulnerable to sea-level rise as well as storm and river flooding due to the low elevation above mean seal level.
